# Adjunctive Hyperbaric Oxygen Therapy in Refractory Crohn's Disease: An Observational Study

**DOI:** 10.1155/2021/6628142

**Published:** 2021-04-26

**Authors:** Marley R. Feitosa, Rogério S. Parra, Vanessa F. Machado, Gustavo N. Vilar, Jussara C. Aquino, José J. R. Rocha, Paulo G. Kotze, Omar Féres

**Affiliations:** ^1^Department of Surgery and Anatomy, Ribeirão Preto Medical School, University of São Paulo, Ribeirão Preto, SP, Brazil; ^2^São Paulo Hospital, Ribeirão Preto, SP, Brazil; ^3^IBD Unit, Health Sciences Postgraduate Program (PPGCS), Pontificia Universidade Católica do Paraná (PUCPR), Curitiba, Brazil

## Abstract

**Background and Aims:**

Patients may experience complications of Crohn's disease (CD) even when treated with optimal medical therapy strategies. Previous data have shown the efficacy of hyperbaric oxygen therapy (HBOT) in the management of complicated CD. However, there is no consensus regarding the optimal number of sessions or duration of treatment regimens. The aim of the present study was to investigate the efficacy of HBOT in CD patients who were refractory to conventional medical management.

**Methods:**

This study included patients who underwent HBOT for the treatment of the following complications: perianal fistulizing Crohn's disease (pCD), enterocutaneous fistulas (ECF), or pyoderma gangrenosum (PG). Complete healing was defined as the closure of external orifice and the absence of active draining (in pCD), complete wound healing (in PG), and granulation or complete wound epithelialization with no enteric draining (in ECF). The persistence of draining and the absence of wound granulation were defined as incomplete healing.

**Results:**

Forty patients were included. The mean CD duration was 10.6 ± 5.8 years. pCD comprised most of the included patients (25/62.5%), followed by ECF (*n* = 13/32.5%) and PG (*n* = 6/15%). In two patients (5%), a combination of ECF and PG was diagnosed, and in one patient (2.5%), all three complications were observed. A total of 32 patients (82.5%) had complete healing. Patients with PG had the highest healing rates (100%), followed by those with ECF (84.6%) and pCD (80%).

**Conclusions:**

Adjunctive HBO was associated with significant healing rates for CD-associated complications such as pCD, ECF, and PG.

## 1. Introduction

Crohn's disease (CD) and ulcerative colitis (UC) are immune-mediated inflammatory conditions characterized by relapsing intestinal inflammation [1]. In CD, the entire gastrointestinal tract may be affected. However, in a significant proportion of cases, the terminal ileum and right colon are involved [2]. Management of CD is usually pharmacological, based on steroids, immunomodulators, and biological agents. The aim of medical therapy in CD is the achievement of clinical remission and mucosal healing [3].

Even with optimal medical therapy strategies, patients may experience disease progression with perianal, intestinal, and extraintestinal complications [4]. These immune and nonimmune complications may present as open wounds such as perianal fistulizing Crohn's disease (pCD), enterocutaneous fistulas (ECF), or pyoderma gangrenosum (PG), an important extraintestinal manifestation of the disease [5–7]. pCD represents a difficult phenotype of the disease that can require intensive medical therapy, wound care, and surgical intervention [8, 9]. PG is usually managed with medical therapy, with different therapeutic options. ECF may develop spontaneously as a consequence of intestinal inflammation or may be a postoperative complication. In both forms, ECF represent a therapeutic challenge, as medical options have limited efficacy and surgery plays an important role as a definitive treatment of these conditions [10].

A proportion of CD patients with pCD, ECF, and PG may develop persistent nonhealing wounds, which represent a clinical and economical challenge [11]. Many associated factors such as hypoxia, alterations in local microbiota, different proinflammatory responses, and dysfunctional tissue remodeling may play an important role in the pathogenesis of these complications [12]. These dysfunctions can be reversed with therapeutic inhalation of 100% oxygen at supra-atmospheric pressures, also known as hyperbaric oxygen therapy (HBOT) [13]. HBOT for refractory CD complications is not a new therapeutic strategy and is used in therapeutic algorithms for more than a decade [14, 15]. Previous data have shown the efficacy of HBOT in the management of complicated CD. Despite its efficacy, there is no consensus on treatment regimens regarding the number of sessions or duration of treatment. The aim of the present study was to investigate the efficacy of HBO on pCD, ECF, and PG in a group of CD patients who were refractory to conventional medical management.

## 2. Methods

### 2.1. Study Design and Data Collection

This was a retrospective study performed using data from a prospectively maintained database from an Outpatient Hyperbaric Center from Ribeirão Preto (São Paulo, Brazil), from January 2008 to December 2019. This database contains the following information from all sessions: patients' identification, medical record number, age at HBO initiation, age at CD diagnosis, CD phenotype (Montreal classification of CD) [16], CD duration, duration of conventional and biological therapy, type of biologic medication, previous intestinal resections, CD-related complications, number of HBO sessions, and clinical evaluation of wound healing.

### 2.2. Population

We included patients who were submitted to HBOT for the following complications: pCD, PG, and ECF. All complications were related to CD activity (pCD or PG) or CD surgery (ECF). Eight patients were excluded due to nonauthorization of sessions by health insurance. Diagnosis and characterization of pCD were confirmed by anorectal examination under anesthesia and pelvic magnetic resonance imaging, which was also used in the follow-up to assess healing. PG was diagnosed by a dermatologist based on clinical history, physical examination, and biopsy to exclude other conditions such as malignancy, infections, or cutaneous vasculitis [17]. Initial assessment of ECF included cross-sectional imaging (computed tomography or magnetic resonance enterography, or pelvic magnetic resonance), and follow-up was carried out clinically. All ECF were postoperative and considered of low output (less than 200 ml/day of intestinal drainage). All patients were refractory to medical therapy, including the use of at least two different antitumor necrosis factor (anti-TNF) agents (infliximab and adalimumab) used in combination with azathioprine in any previous phase of their disease course. In addition, anti-TNF optimization was performed through clinical parameters, since therapeutic drug monitoring (TDM) is not available in our country, comprehensively. This current study is an extension of the information presented in the earlier article published by the same team [15].

### 2.3. HBOT Sessions and Healing Definition

HBOT sessions were held at a 2800 Sechrist Monoplace Hyperbaric Chamber™ (Sechrist, USA), pressurized to 2.4 ATA, with duration of 2 hours. The initial protocol included 40 daily and consecutive sessions; however, after 10 sessions, patients were clinically reassessed, by a hyperbaric physician and an inflammatory bowel disease (IBD) specialist, who could decide to continue or to interrupt HBOT treatment. Therefore, the number of sessions varied according to clinical improvement. Complete healing was defined as the closure of external orifice and the absence of active draining (in pCD), complete wound healing (in PG), and granulation or complete wound epithelialization with no enteric draining (in ECF). The persistence of draining and the absence of wound granulation were defined as incomplete healing. If complete healing was observed, the treatment was interrupted. In cases with partial healing after 40 sessions, HBOT was prolonged until complete healing with variable duration of sessions. In nonresponders after 40 sessions, HBOT was discontinued. Basic wound care of chronic cutaneous lesions was maintained throughout the entire treatment regimen and included cleaning and dressings, antibiotics, and surgical debridement whenever needed (except for PG). Loose setons were used in actively draining perianal fistulas.

### 2.4. Adverse Events

Adverse events such as ear barotrauma, seizure, claustrophobia, and lung barotrauma were observed and tabulated at the end of each session, should they occur. The monitoring of these adverse events was carried out by the nurse and hyperbaric doctor.

### 2.5. Ethics Approval

This study was approved by the Institutional Review Board and the Ethics Committee (no. 07/2020, 02. April.2020). All patients signed an informed consent when HBOT was initiated, allowing retrospective data collection. All procedures were in accordance with the 1964 Helsinki declaration and its later amendments or comparable ethical standards.

### 2.6. Statistical Analysis

Statistical analysis was performed with IBM SPSS statistics version 20.0 (SPSS, Chicago, IL, USA). Categorical variables were expressed as absolute and relative frequencies. Continuous variables were expressed as mean ± standard deviation (SD). The Kruskal-Wallis test was used to check for normality of variables. The mean number of HBOT sessions was compared among indications, and significance was calculated with the ANOVA test. Comparison of healing rates was performed in a three-way crosstab, and significance was calculated by the Pearson chi-square test. *P* values inferior to 0.05 were considered statistically significant.

## 3. Results

Overall, 40 patients with refractory CD with equal gender distribution were included in the analysis. Patients' baseline characteristics are described in detail in Table 1. The mean age at treatment initiation was 38.0 ± 12.7 years (range 11-76 years). The mean CD duration was 10.6 ± 5.8 years. According to Montreal classification, most patients were diagnosed with CD between 17 and 40 years of age (A2, *n* = 36, 90%) and had ileocolic disease (L3, *n* = 28, 70%) and nonstricturing nonpenetrating behavior (B1, *n* = 19, 47.5%). pCD was diagnosed in 25 (62.5%) patients, representing the most common indication for HBOT, followed by ECF (*n* = 13/32.5%) and PG (*n* = 6/15%). In two cases (5%), a combination of ECF and PG was diagnosed, and in one case (2.5%), all three complications were observed. Mean duration of anti-TNF treatment was 29.1 ± 16.6 months.

**Figure 1:**
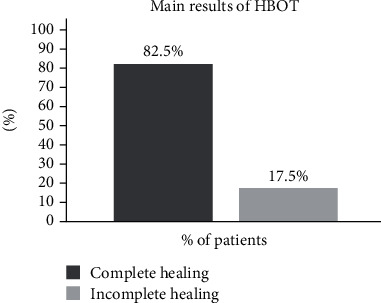
Main results of HBOT in 40 patients.

**Figure 2:**
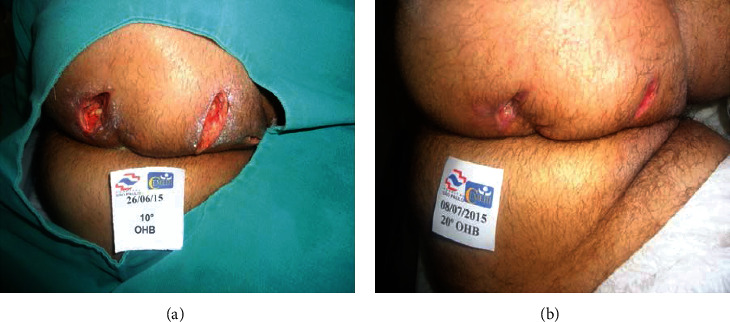
Significant healing of perineal fistulizing Crohn's disease wounds observed at the beginning of treatment (10 sessions) after perineal surgery (a) and at the end of 20 sessions (b).

**Figure 3:**
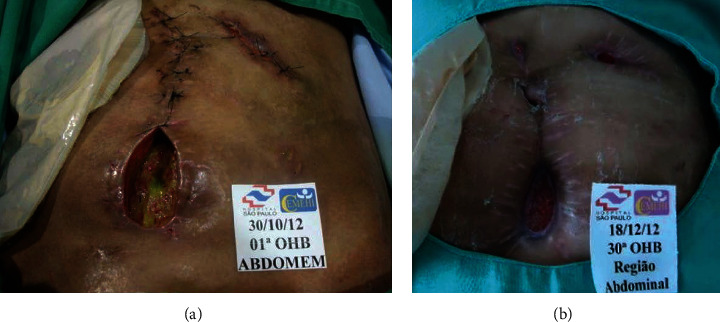
Enterocutaneous fistula with active draining at the beginning of hyperbaric oxygen therapy (a). After 30 sessions, closure of the fistula tract was observed (b).

The average number of sessions was 29.1 ± 16.6 (range, 10-86). Overall, 1166 sessions were performed and no complications related to HBOT were observed, such as claustrophobia and ear barotrauma. A total of 33 patients (82.5%) had complete healing of the complications, and 7 (17.5%) presented incomplete healing (Figure 1). No difference was observed in age at CD onset and age at HBOT initiation, CD duration, duration of anti-TNF therapy, and mean number of sessions and healing rates between the 3 different indications (pCD, ECF, or PG).  Table 2 summarizes patients' characteristics and healing rates according to the indication for HBOT. In summary, PG had the highest healing rates (100%), followed by ECF (84.6%) and pCD (80%). The clinical effects of HBOT are illustrated in Figures 2 and 3 (two examples of cases). There were no adverse events related to HBOT.

## 4. Discussion

In this study, we present the largest number of CD cases treated with HBOT and published in the English language [12, 18]. We observed that adjunctive HBOT was associated with high rates of complete healing of CD-related complications, such as pCD, EFC, and PG without the need for invasive surgical procedures and with adequate safety, as no HBOT-related adverse events were observed. There is growing interest in the application of HBOT in patients with IBD. This therapeutic option has been commonly used in chronic wounds, decompression sickness, diabetic foot, Fournier's gangrene, and carbon monoxide poisoning [19]. Recently, it has been used in emerging indications such as CD-associated complications [12, 19]. In vitro and in vivo studies have demonstrated that hypoxia is an important factor that triggers and maintains inflammation [20, 21]. HBOT drives more oxygen into injured tissues, a first measurable effect and a signal for a complex cascade of events that will ultimately lead to microbiome changes, modulation of the immune system towards an anti-inflammatory state, and improved healing in IBD patients [22]. In fact, HBOT has been shown to be an effective adjuvant therapy for steroid-refractory UC, perianal CD, PG, and delayed perineal wound healing after proctectomy in CD [20, 23].

pCD is a predictor of poor prognosis in CD patients [24, 25]. Up to 28% of CD patients may develop perianal fistulas after 20 years of diagnosis [8]. In addition to the high frequency, this phenotype of CD is usually associated with purulent discharge, multiple surgical interventions, risk of proctectomy, and permanent stomas, with a significant impact on patients' quality of life [8, 26]. In the present study, all cases were refractory to at least two anti-TNF agents, used in combination with antibiotics, azathioprine, and loose setons for more than 6 months. In this setting, therapeutic options are limited and include the following: (I) switch for biologics with different mechanisms of actions (i.e., vedolizumab or ustekinumab), despite limited evidence for both agents in the pCD scenario [8]; (II) switch of the immunosuppressive agent for tacrolimus or cyclosporin (prospective data is scarce, and high rates of relapse are observed after drug discontinuation [8]); or (III) surgery (temporary or permanent stoma with proctectomy), a procedure with serious impact on long-term quality of life, especially in young patients [8, 27]. In our clinical practice, major abdominal surgery involving stomas is reserved for a limited number of cases, with refractory perianal sepsis and incontinence. The challenges of current therapeutic options in refractory pCD patients make HBOT an attractive alternative, as it is usually a safe treatment [12]. In the current series, no adverse events related to HBOT were observed.

Several factors can prevent healing in pCD [28]. HBOT can act in situations that may contribute to persistence of active fistulizing disease. First, the increase in tissue oxygenation creates a highly unfavorable environment for bacterial overgrowth [29]. Local fistula microbiome may differ from that observed in the intestinal lumen and has been considered to promote and maintain inflammation within the fistula tract [30]. Several studies have observed the accumulation of macrophages, lymphocytes, and proinflammatory cytokines on the wall of fistula tracts. On the other hand, anti-inflammatory properties have been demonstrated with HBOT [12]. Lastly, wound repair failure has been observed in fistulizing disease and HBOT studies have demonstrated that increases in blood flow and in reactive oxygen concentration may promote healing even in the absence of myofibroblast proliferation and fibroblast recruitment [31]. Wound contraction, collagen deposition, and reversion of epithelial-mesenchymal transition may play a role in HBOT-treated pCD regeneration tissue [31]. These factors can explain the efficacy of HBOT in IBD patients.

Although rare, spontaneous ECF are challenging manifestations of CD, as they are a marker of advanced transmural disease and are usually encountered in malnourished patients. In our study, ECF developed after intestinal resections and there was no disease activity at the anastomotic site or in other segments of the small bowel or colon. We believe that malnutrition and inflammation were the impending factors for ECF closure. In fact, inflammation appears to play an important role even in non-CD EFC. A study by Rahbour et al. [32] observed ongoing production of TNF-alpha in non-CD tissues collected from surgical specimens of EFC. In our experience, most CD-related ECF will have to undergo abdominal surgery after improvement of nutritional status, as there is limited efficacy in ECF closure with biological agents. However, if the intestinal wall is not affected by active CD inflammation, a combination of HBOT and traditional medical management [10], including bowel rest, artificial nutrition, rehydration and correction of electrolyte disturbances, sepsis control, somatostatin analogues, and proper wound care, may represent an interesting and more conservative option and, in our study, prevented abdominal surgery in all cases. Of note, local unfavorable conditions that prevent ECF closure include short (<2 cm) and multiple tracts, high output (>500 ml), associated bowel inflammation, adjacent infection, and distal obstruction. These factors constitute predictors of surgery in ECF (CD-related or not) and were always assessed and excluded in our patients before HBOT was recommended [33]. Another interesting information regarding HBOT and inflammation in CD patients is that some level of improvement in small bowel inflammation was evidenced on follow-up magnetic resonance enterography, in some patients. This finding may be a consequence of a reduction in local inflammation and in bowel oedema. Also, wound contraction and tissue remodeling with collagen deposition are usually observed in HBOT-treated patients [31] and, in our series, all patients were discharged with closed abdominal fistulas.

Regarding PG, its association with IBD is not uncommon [34]. Clinical hallmarks of this skin condition include the following: progressive ulcer of irregular shape, undermined and reddish-violaceous borders [35]. Multiple lesions may occur [35]. PG may appear in cutaneous trauma sites and improvement of lesions may be observed with immunosuppressant therapy [35, 36]. Management of severe PG, as in our cohort, requires a combination of efforts. Basic measures include pain and infection control, adequate local wound care, systemic therapy (e.g., corticosteroids), and targeted therapy (e.g., anti-TNF agents or anti-interleukin-12 and 23) [9]. Most PG-associated lesions will heal with standard care. However, some factors may prevent ulcers from healing. Since all patients in our cohort had no signs of luminal CD activity, we hypothesized that local inflammation and changes in microbiota may have played a role in difficult healing. PG itself may be considered an inflammatory disease [35], and the effective anti-inflammatory properties combined with angiogenesis and tissue remodeling [37] may have explained the successful reports achieved with HBOT in our PG cases and in others [38–40].

Overall, scientific evidence that HBOT is associated with positive effects in IBD patients is, at most, of moderate quality. In a systematic review of 17 studies involving 613 patients with IBD, Dulai et al. [12] recognized the potential and safety of HBOT in the management of IBD patients. However, the scarcity of well-controlled randomized trials makes it impossible to understand the effects of this therapy on the natural history of the disease. The same author conducted a prospective trial to elucidate the role of HBOT on hospitalized UC patients. HBOT was associated with higher clinical remission, less progression to second-line therapy during hospitalization, and lower rates of in-hospital colectomy [41]. However, conclusions were underpowered as the study was terminated early due to poor recruitment. More recently, the HOT-TOPIC trial has been registered in the Netherlands. The study has two coprimary endpoints: changes in the perianal disease activity index and MRI scores [42]. Valuable data is expected.

Despite the valuable information presented, our study is associated with some limitations that need to be addressed. This is a retrospective analysis, with information extracted from a prospectively maintained database, with a single group of patients with no control group for comparisons. Included patients had different complications of CD in the same cohort (pCD, PG, and ECF) and did not represent a homogeneous population of patients. Our included patients had postoperative and not spontaneous CD-related ECF. In addition, MRI was not performed in all patients after HBOT session. Results of our study are encouraging since a significant number of real-world clinical practice patients achieved complete healing of CD complications with no adverse events and without major abdominal surgeries. However, it is important to emphasize that the effect of HBOT occurs during treatment, with good early response. The long-term effects are not fully known. Success in healing cannot be attributed exclusively to close monitoring of patients (which included daily clinical evaluations, intensive wound care, and optimization of medical therapy) because all patients were refractory to standard care.

In summary, adjunctive HBOT was associated with successful healing rates in CD-associated complications such as pCD, ECF, and PG. Randomized controlled trials in early and late-stage CD patients are warranted to increase knowledge on the specific role of HBOT in daily IBD practice.

## Figures and Tables

**Table 1:** Baseline characteristics of patients and treatment.

Characteristic	*N* = 40
Gender	
Male	20 (50%)
Female	20 (50%)
Age at HBOT	
Mean ± SD (years)	38.0 ± 12.7
CD duration	
Mean ± SD (years)	10.6 ± 5.8
Montreal classification	
Age at CD onset	
A1 (below or equal to 16 years)	1 (2.5%)
A2 (17 to 40 years)	36 (90%)
A3 (>40 years)	3 (7.5%)
Disease location	
L1 (terminal ileum)	8 (20%)
L2 (colon)	4 (10%)
L3 (ileocolon)	28 (70%)
Disease behavior	
B1 (nonstricturing nonpenetrating)	19 (47.5%)
B2 (structuring)	8 (20.0%)
B3 (penetrating)	13 (32.5%)
HBOT indication	
pCD	24 (60%)
ECF	10 (25%)
PG	3 (7.5%)
ECF+PG	2 (5%)
ECF+pCD+PG	1 (2.5%)
Previous bowel resections	19 (47.5%)
Medical therapy (at HBOT)	
Anti-TNF agents	40 (100%)
Azathioprine	40 (100%)
Corticosteroids	3 (7.5%)
Duration of anti-TNF therapy (at HBOT)	
Mean ± SD (months)	42.0 ± 37.5
Number of HBOT sessions	
Mean ± SD	29.1 ± 16.6

HBOT: hyperbaric oxygen therapy; SD: standard deviation; pCD: perineal fistulizing Crohn's disease; ECF: enterocutaneous fistula; PG: pyoderma gangrenosum.

**Table 2:** Patients' characteristics and HBOT effect according to indication.

Characteristic	pCD	ECF	PG	*P*
Age (at CD onset)				
Mean ± SD (years)	27.8 ± 10.1	26.1 ± 6.5	29.1 ± 9.2	0.87^∗^
Age (at HBOT)				
Mean ± SD (years)	38.3 ± 13.7	37.2 ± 11.1	41.0 ± 12.8	0.83^∗^
CD duration				
Mean ± SD (years)	10.5 ± 6.2	11.8 ± 5.7	11.8 ± 3.1	0.95^∗^
Duration of anti-TNF therapy				
Mean ± SD (months)	42.8 ± 21.6	43.6 ± 16.8	36.1 ± 11.5	0.81^∗^
Number of HBOT sessions				
Mean ± SD	29.9 ± 15.0	30.8 ± 21.5	29.3 ± 12.7	0.98^∗^
Complete healing rate				
*N* (%)	20 (80.0)	11 (84.6)	6 (100.0)	0.38^∗∗^

pCD: perineal fistulizing Crohn's disease; ECF: enterocutaneous fistula; PG: pyoderma gangrenosum; CD: Crohn's disease; SD: standard deviation; HBOT: hyperbaric oxygen therapy. ^∗^ANOVA test. ^∗∗^Pearson chi-square test.

## Data Availability

Data were collected by the computerized hospital system and medical records. All data analyzed during this study are included in this published article.

## Abbreviations

CD: Crohn's disease
ECF: Enterocutaneous fistulas
HBOT: Hyperbaric oxygen therapy
IBD: Inflammatory bowel disease
pCD: Perianal fistulizing Crohn's disease
PG: Pyoderma gangrenosum
TNF: Tumor necrosis factor
UC: Ulcerative colitis.

## Additional Points

*Key Summary*. *Current Knowledge* Even when treated with optimal medical therapy strategies, Crohn's disease patients may experience complications. A proportion of patients with perianal fistulizing Crohn's disease, enterocutaneous fistulas, and pyoderma gangrenosum may develop persistent nonhealing wounds, which represent a clinical and economic challenge. Previous data have shown the efficacy of hyperbaric oxygen therapy in the management of complicated Crohn's disease patients. The majority of published studies involved case reports or small case series, and there is no consensus regarding the optimal number of sessions or duration of treatment for treatment regimens. *New Findings of This Study*. This study comprises the largest number of Crohn's disease cases treated with hyperbaric oxygen therapy published in the English language. Adjunctive hyperbaric oxygen therapy was associated with significant rates of complete healing of Crohn's disease-related complications with no need for invasive surgical procedures and with adequate safety. Hyperbaric oxygen therapy for refractory Crohn's disease complications is a therapeutic strategy that could be more used in treatment algorithms.
